# Potential biological contributers to the sex difference in multiple sclerosis progression

**DOI:** 10.3389/fimmu.2023.1175874

**Published:** 2023-04-14

**Authors:** Nuria Alvarez-Sanchez, Shannon E. Dunn

**Affiliations:** ^1^Keenan Research Centre for Biomedical Science, St. Michael’s Hospital, Toronto, ON, Canada; ^2^Department of Immunology, 1 King’s College Circle, Toronto, ON, Canada; ^3^Women's College Research Institute, Women's College Hospital, Toronto, ON, Canada

**Keywords:** sex difference, multiple sclerosis, neurodegeneration, T cells, glia, mitochondria, microbiota

## Abstract

Multiple sclerosis (MS) is an immune-mediated disease that targets the myelin sheath of central nervous system (CNS) neurons leading to axon injury, neuronal death, and neurological progression. Though women are more highly susceptible to developing MS, men that develop this disease exhibit greater cognitive impairment and accumulate disability more rapidly than women. Magnetic resonance imaging and pathology studies have revealed that the greater neurological progression seen in males correlates with chronic immune activation and increased iron accumulation at the rims of chronic white matter lesions as well as more intensive whole brain and grey matter atrophy and axon loss. Studies in humans and in animal models of MS suggest that male aged microglia do not have a higher propensity for inflammation, but may become more re-active at the rim of white matter lesions as a result of the presence of pro-inflammatory T cells, greater astrocyte activation or iron release from oligodendrocytes in the males. There is also evidence that remyelination is more efficient in aged female than aged male rodents and that male neurons are more susceptible to oxidative and nitrosative stress. Both sex chromosome complement and sex hormones contribute to these sex differences in biology.

## 1 Introduction

Multiple Sclerosis (MS) is a chronic, immune-mediated, demyelinating disease of the central nervous system (CNS) that affects 2.8 million people world-wide (1). MS most frequently debuts as a relapsing-remitting disease (called relapsing-remitting MS or RR-MS) that is characterized by relapses followed by periods of partial to complete neurological recovery (1). Without treatment intervention, the majority of people with RR-MS will transition to having a progressive course, called secondary progressive (SP)-MS, within a few decades of diagnosis (2). Less frequently, patients exhibit a progressive trajectory of disability from onset, which is termed primary progressive (PP)-MS (1). Although indistinguishable from SP-MS according to the major MS risk genes or the nature of the CNS pathology, PP-MS has a later age of onset and white matter (WM) lesions in PP-MS tend to be less inflammatory and exhibit more myelin and axon damage than in SP-MS (3, 4).

Relapsing-onset MS has a strong female prevalence, which is thought to be a result of females having more robust T cell-mediated autoimmunity [reviewed in (5)]. By contrast, males if they develop MS, are more susceptible to neurodegeneration in this disease. Natural history studies of untreated MS patients have shown that males with RR-MS also have a higher risk of developing SP-MS and demonstrate a shorter time to conversion to this phase of disease compared to female counterparts (6–8). Males with MS also exhibit more extensive whole brain and grey matter (GM) atrophy and ventricular enlargement compared to females indicating more extensive neuronal loss in males (9–11). The underlying mechanisms for the male predilection for neurodegeneration and disease progression are not well understood. Here, we overview the evidence that males with MS exhibit more rapid neuron deterioration than females with this disease and describe how MS pathology differs between men and women to account for these differences. We further discuss the underlying cellular mechanisms of the male predilection for CNS injury drawing from both studies in human MS and animal models of this disease.

## 2 Male sex is associated with more rapid progression and CNS tissue loss in MS

### 2.1 Neurological progression and cognitive decline are more rapid in males with MS

There is strong evidence that males are more prone to neurological deterioration than females with MS. Males exhibit greater deficits in finger dexterity as measured using the 9 hole peg test (12, 13) and exhibit worse walking and disability scores (14, 15) compared to females. Postural stability is also reduced more so in men than women with progressive forms of MS (16). Men also have a lower odds of developing a benign course of MS (17–19), and some cohort studies report that men with MS experience greater mortality than women (20–23). Natural history studies that have tracked the progression of relapsing-onset MS in untreated MS populations have reported that men are at higher risk for development (8) and have a shorter time to conversion to SP-MS (7, 24, 25). Males are more likely than females to debut with PP-MS and men with this form of MS exhibit a more rapid progression of disability when compared to female counterparts (26).

Male sex is also a predictive factor for having a shorter time to reaching a disability milestone of expanded disability status scale (EDSS)3 and EDSS6 (25, 27–29); EDSS3 is a stage when patients show mild to moderate disability, but do not have mobility issues, whereas EDSS6 is a stage where walking is affected and supports such as a cane are required for ambulation. Interestingly, this effect of male sex in hastening the onset of SP-MS and disability accrual has not been observed in more recent studies where a higher proportion of MS patients are receiving high-efficacy disease modifying therapies (30, 31), suggesting that the autoimmune inflammation may be a factor in the effect of male sex on progression.

Individuals with MS also experience cognitive deficits including reductions in verbal and non-verbal memory, information processing speed, attention, and executive functioning (32–36). A large number of studies have reported that males are more cognitively impaired than females across a number of these domains (10, 32, 34, 36–43). A sex difference in cognition has also been observed in newly diagnosed MS patients, where males show increased anxiety and depression scores, and poorer attention compared to females (34). One study reported that the greater cognitive deficits seen in males with early MS correlated with more extensive white matter damage, particularly in the thalamus, which is a critical relay center in the brain (10). Males with MS also experience more severe deficits in processing speed during relapses compared with females, indicating that individual immune attacks may be more detrimental to the CNS in males (44).

A number of studies have also assessed differences in functional connectivity and neuronal network efficiency in the brains of men and women with MS using functional magnetic resonance imaging (fMRI) and other MRI-based approaches. For example, Schoonheim et al. (2012) investigated the extent of synchronization of activation of different brain regions in the resting state using fMRI (42). MS patients were matched for educational status, pre-morbid intelligence, disability, disease duration, and extent of white matter damage as assessed by T2 lesion load (42). It was found that functional connectivity and network efficiency were lower in the male compared to the female MS patients and that these deficits correlated with worse performance on a visuospatial memory task in males (42). Similarly, a large study of MS that assessed hippocampal volumes and networks using an MRI-based approach reported that, compared to males, females exhibited a more clustered hippocampal network topology (i.e., increased local connectivity in the hippocampus) that correlated with better cognitive and memory performance (45). In contrast, a study of patients at an earlier stage of MS reported that functional connectivity between temporal and inferior frontal lobe areas and the right amygdala and right olfactory cortex was higher in males than in females, correlating positively with a greater extent of GM atrophy in specific brain areas in the males (46). In this case, it was speculated that the increased functional connectivity was an early compensatory mechanism to retain cognitive performance in the males (46). In conclusion, the brain functionally reorganizes its neuronal circuits to maintain cognitive function in response to CNS damage in MS. This may occur more so in men early in disease in response to the increased tissue damage in this sex, however as the disease progresses, females may have a better reserve for functional re-organization as a result of preserved neuronal function.

### 2.2 Post-mortem studies and MRI studies of those living with MS suggest that inflammation persists and neurodegeneration is more severe in males

Pathology studies in post-mortem brain samples in MS have been crucial to understanding underlying pathology in MS. These studies have elucidated that the focal inflammatory demyelinating lesions in the WM that underlie the acute attacks in early MS contain CD4^+^ and CD8^+^ T cells, activated macrophages and microglia, and show evidence of active demyelination and axonal transection and injury (47). Some of these studies examined sex differences, but did not detect any differences in either the number or proportion of acute lesions or in the numbers of T cells or activated macrophages/microglia within male or female WM lesions (48–50). Notwithstanding, these studies did not conduct a deep immunophenotyping of immune cells within the WM lesions in men and women.

With aging of MS, peripheral immune-mediated inflammation subsides and WM lesions evolve either into *inactive* plaques that only have sparse immune cells present or alternatively *smoldering* lesions that have a demyelinated core with a rim of activated microglia along the lesion edge in close proximity to lymphocytic perivascular cuffs (alternatively referred to as chronic-active or mixed active/inactive lesions). Inactive lesions can be either chronically-demyelinated or myelinated (also called shadow plaques) (47). Of these lesion types, it is the proportion of smoldering lesions that associates with the development of a more severe MS disease course (51). In this regard, two large post-mortem studies of CNS specimens reported finding a higher proportion of smoldering lesions at the expense of inactive lesions in male compared to female MS brain specimens (49, 50), suggesting that immune-mediated inflammation persists longer in WM lesions in males.

Quantitative susceptibility mapping (QSM) is an advanced MRI method that is used to examine tissue concentrations of paramagnetic iron, which accumulates in activated microglia at the rim of smoldering lesions. A small study of QSM in living MS patients reported that male sex conferred a 10-fold higher risk of having more than one lesion with an active rim, even after adjusting for age, symptom duration, EDSS, and MS subtype (52). These findings are consistent with there being more activated microglia at the rims of classic WM lesions in men compared with women living with MS. Whether this increased microglia activation in male WM lesions is driven by mechanisms intrinsic to microglia, increased brain iron load in the males, or to immune products released from T or B cells that reside in the perivascular spaces in these areas remains unknown.

In addition to classic WM lesions, demyelination occurs in the cortices in MS and these demyelinated plaques are referred to as cortical lesions. Cortical lesions are evident even in the earliest stages of MS, but increase in number as the disease progresses (53). In this regard, a post-mortem study of advanced MS reported that males with MS exhibited a higher frequency of cortical lesions compared to females with this disease (49). Similarly, MRI studies have reported detecting a higher number of intracortical lesions in male versus females living with MS (54). The presence of these lesions correlated with EDSS, lesion load, and the extent of brain atrophy in the patients (54), suggesting that the increased presence of cortical lesions may be an additional contributor to the more severe disability seen in males with MS.

As MS progresses, there is an increase in the proportion of chronically-demyelinated lesions at the expense of myelinated lesions (55). This loss of myelin is associated with reduced axon conduction, an increase in the metabolic cost of action conduction, and an increased vulnerability of axons to inflammatory and oxidative injury (56). Chronic demyelination in MS results either from an age-related decline in oligodendrocyte precursor cells (OPCs) or a reduced ability of these cells to differentiate into mature oligodendrocytes (mOL) in the environment of the WM lesions due to the presence of pro-inflammatory cytokines and myelin inhibitory proteins (57, 58). To date, pathological studies in MS have not detected any differences in the number of OPCs or mOL in male and female MS brain specimens (48). The proportion of shadow plaques also does not differ between male and female MS specimens (49, 50, 59); albeit, one study noted a trend for females having a greater proportion of shadow plaques as compared to males during middle-age (50). A smaller study of MS specimens that scored the extent of myelination in shadow plaques reported finding a trend for a higher myelination scores in early WM lesions in female MS specimens (55). Together, these findings suggest a possible tendency for greater preservation of myelin in MS plaques in females.

The inflammation and demyelination that occurs in both WM and cortical lesions leads to axon injury and loss, which is considered to be the major factor leading to disability and cognitive deficits in MS (60, 61). A small post-mortem study that examined axon density at the level of the cervical and thoracic spine reported that the males exhibit greater spinal cord atrophy and reduced axon density compared to females with MS (62). Tissue damage can be also estimated in WM lesions of living patients using advanced MRI techniques such as diffusion tensor imaging (DTI). Consistent with pathology findings, DTI studies have reported the presence of more extensive WM damage in males compared with females with MS (37, 63). One of these studies that matched male and patients for WM lesion volumes, disease duration, and disability scores, showed that males exhibited more WM damage in the deep grey matter structures including the thalamus (37). Cerebrospinal fluid (CSF) levels of neurofilament light chain, a marker of axonal damage, are also reported to be higher in males compared with females with MS, especially in those with PP-MS (64, 65).

Atrophy of the whole brain and GM, which contains the cell bodies of neurons, also occurs in MS and the extent of GM atrophy is a predictor of cognitive and disability progression in this disease (66). With some exceptions (9, 67), most studies report that the extent of whole brain (11, 68, 69) and GM atrophy (9–12, 37, 70) is greater in male than female patients. In addition, one study that evaluated cortical volumes in MS by MRI reported that cortical atrophy is also more extensive in male compared to female patients, but only when the studied females were restricted to those having a pre-menopausal MS onset (68), pointing to ovarian hormones as protective factors against neurodegeneration in MS. In this regard, there is evidence that both testosterone and estrogens protect against neurodegeneration in MS (71).

Optical coherence tomography is a technique that has been used to measure the thickness of the retinal nerve fiber layer. The retinal nerve fiber layer contains the axons of retinal ganglion neurons that continue through the optic nerve, which is targeted by the autoimmune process in MS. Studies in pediatric MS (<18 years) showed that there is more advanced retinal nerve fiber layer thinning in males compared to females with MS, suggesting that sex differences in neuronal vulnerability may be apparent even in children with this disease (72). Taken together, these studies have revealed a greater persistence of inflammation in WM lesions and a greater cortical lesion burden in men compared to women with MS as they age. This is accompanied by more severe axon loss and whole brain and GM atrophy in men. These findings lie in sharp contrast with the knowledge that the likelihood of MS and the frequency of relapses is higher in females with early RR-MS [reviewed in (5)], suggesting that autoimmune inflammation either unfolds differently in the CNS of men vs. women or that male neurons or myelin are more vulnerable to inflammatory insults.

## 3 Cellular mechanisms that have the potential to contribute to increased axonal and neuronal injury in MS

Although the mechanisms of axonal and neuronal loss in MS are not completely understood, it is known that neuronal injury is initially caused by the inflammation associated with the development of autoimmune T cell attacks (73). Early acute MS lesions contain transected axons and the extent of this transection correlates with the number of immune cells present in lesions (74). T cell and monocyte infiltration in the CNS also leads to microglia activation. Activated microglia and peripheral immune cells together produce pro-inflammatory cytokines, reactive oxygen species (ROS), and reactive nitrogen species (RNS), which lead to the development of glutamate excitotoxicity in neurons (75, 76). This oxidative stress and glutamate excitotoxicity leads to mitochondrial dysfunction in the neurons (75, 76). In this regard, mitochondria in the deep cortical neurons in the progressive MS brain exhibit impaired mitochondrial respiratory activity (77), increased mitochondrial DNA mutations, and defects in proteins associated with the mitochondrial respiratory chain complex (78, 79). Inefficiencies in the respiratory chain complex lead to increases in mitochondrial ROS, which can further compound oxidative damage (80). Mitochondrial dysfunction also triggers the activation of calcium-dependent degradation pathways within neurons (81). The loss of OLs in lesions leaves axons more vulnerable to the effects of inflammation and ROS and increases the energy requirements for maintaining the membrane potential, further taxing mitochondria in the neurons (56). OLs have been also shown to support neurons metabolically by supplying them with lactate (82, 83), and loss of this support causes degeneration both *in vitro* and *in vivo* (82). Pro-inflammatory microglia also induce the acquisition of subtypes of astrocytes (e.g. A1-like astrocytes) that have neurotoxic activity (84). Certain neurons appear to be particularly vulnerable to this inflammation and oxidative stress, including the excitatory CUX2^+^ neurons in the upper cortical layers in the brain (85), inhibitory (86) or parvalbumin-positive interneurons (87), and corticospinal neurons (88).

Though there is a correlation between WM lesion burden and disability in early MS, this relationship becomes uncoupled in more advanced MS (89). This is because T cell and microglia activation are no longer restricted to WM lesions and inflammation spreads into the “so-called” normal appearing white matter (NAWM) and normal appearing grey matter (47). In addition, cortical demyelinating lesions become more prominent contributors to overall axon injury (47). Further, the axon transection that occurs in WM lesions initiates a process of anterograde and Wallerian degeneration of the neurons that continues past the lesion site (90). Disruption of axon transport in injured axons also leads to atrophy of the neuron soma and degeneration of synapses, which disrupts transmitter and trophic support (90). This can lead to the loss of neighbouring neurons (a process called trans-synaptic degeneration) (90), which may also explain why relay centers such as the thalamus are susceptible to atrophy in MS. Therefore, neurodegeneration in MS is a complex process that is initiated by inflammation, ROS and RNS production, and glutamate excitotoxicity that leads to demyelination, axon transection and injury, mitochondrial stress, a gradual dying back of neurons, and transynaptic nerve degeneration. The increased neuronal damage in males with MS may relate to sex differences in any one of these factors or to sex differences in the brain’s endogenous repair mechanisms. How male sex can alter these pathological processes will be discussed in the following sections.

## 4 Male T cells on an individual basis may be more pro-inflammatory in MS and EAE

Experimental autoimmune encephalomyelitis (EAE) is an immune-based model of MS that is most commonly induced in rodents by immunization with protein components of the myelin sheath and adjuvants such as Complete Freund’s adjuvant (CFA) and pertussis toxin (called active EAE) (91). Immunization with myelin protein induces the expansion and differentiation of myelin-specific T helper 1 (Th1) and T helper 17 (Th17) cells that migrate to the spinal cord and brain to initiate inflammatory demyelination. As in MS, EAE is characterized by T cell and monocyte infiltration, ROS and RNS production, glutamate excitotoxicity, and synapse loss, leading to focal mitochondrial and axon damage (92, 93). Over time, this axon damage progresses to neuron loss and spinal cord atrophy (94, 95). Despite the utility of EAE in modeling these aspects of MS progression, studies in conventional EAE have not been successful in modeling the effect of male sex in accelerating neurological deterioration. This is because the pathology in these models is dominated by Th-driven inflammation, and myelin-specific Th cells have a greater propensity to expand and acquire a pro-inflammatory Th1 phenotype in female mice [reviewed in (5)].

However, an effect of male sex in increasing EAE severity has been observed in passive EAE experiments where an equivalent amount of purified male or female myelin-specific T cell receptor (TCR) CD4^+^ T cells are used to induce the disease (96–98). For example, when myelin-oligodendrocyte glycoprotein (MOG) TCR transgenic T cells (NOD background) are activated *in vitro* under Th1 or Th17 polarization conditions and then transferred into NOD.SCID recipients, male MOG-specific TCR T cells induce a more progressive EAE course compared to female T cells, that associates with a greater propensity of the donor MOG-reactive Th17 cells to produce interferon (IFN-)γ (96). Gonadectomy in mice confirmed that this sex difference was not due to gonadal hormone influences (96). Studies that used the four core genotype mouse model, which segregates the influence of sex hormones from sex chromosomes, clearly showed that the more progressive EAE phenotype in the male TCR transgenic T cells was due to lowered expression of Jarid1c, an X-chromosome encoded-histone demethylase. T cells isolated from male MS patients also exhibited reduced Jarid1c expression compared to female T cells (96), indicating that this is a mechanism that is conserved in mice and humans.

Consistent with these findings, a study that used the 2D2 line of MOG TCR transgenic mice (C57BL6/J background) showed that male TCR transgenic T cells that were polarized to Th1 were more encephalitogenic than female Th1 cells upon passive transfer. In this case, the more severe EAE correlated with a higher frequency of the male MOG-specific TCR Tg Th1 cells acquiring a memory effector phenotype upon *in vitro* activation with MOG peptide and interleukin (IL) -12 (97). Another study, that conducted transfers using MOG TCR transgenic T cells from 1640 mice on the SJL background, showed that male TCR transgenic T cells caused a more chronic form of EAE compared to the females, which instead showed a relapsing-remitting EAE phenotype. In this case, the chronic EAE phenotype elicited by the male T cells associated with reduced expression of genes involved in T regulatory (Treg) differentiation and reduced Treg clustering in the CNS lesions (98). These findings are consistent with the finding of a higher frequency of Treg in females compared to males with clinical isolated syndrome that later fulfilled the diagnosis criteria for MS (99). Together, these studies suggest that male murine myelin-reactive Th effector cells are more pathogenic than female Th cells and are skewed more towards an effector phenotype and away from a regulatory T cell phenotype.

While CD4^+^ T cells are the major players in EAE pathology and MS initiation, CD8^+^ T cells feature more prominently in WM and intracortical lesions in MS (47, 100) and viruses such as Epstein Barr Virus (EBV), which evoke CD8^+^ T cell immunity, have been implicated in MS initiation (101). CD8^+^ T cells are present in the perivascular cuffs in MS lesions and in the NAWM, are clonally-expanded, and exhibit a tissue-resident, effector memory phenotype characterized by high expression of activation (CD69, Ki67, ICOS) and cytotoxic (FasL, granzyme B) markers and pro-inflammatory cytokines (IFN-γ, tumor necrosis factor (TNF)) (51, 102). Though it is controversial whether EBV transcripts are present in MS lesions (103), lesional CD8^+^ T cells can be cross-activated by EBV antigens *ex vivo* (102). This has led to the hypothesis that EBV plays a role in the initial activation of the autoreactive CD8^+^ T cells that then home to the CNS where they recognize CNS antigens (102). However, it remains to be elucidated whether EBV-specific or autoreactive CD8^+^ T cells differ in phenotype between men and women and contribute to differences in inflammation or axonal injury in MS lesions.

What is known is that CD8^+^ T cells of healthy females have a greater capacity to expand and are more likely to adopt a T effector phenotype compared to male CD8^+^ T cells, characterized by high IFN-γ and granzyme B production (104). This more robust CD8^+^ T cell effector activity is associated with greater overall anti-viral immunity in females (104). In this regard, there is evidence that slower viral clearance in males can lead to increased MS or CNS autoimmunity in animal models. For example, polymorphisms in the cytolytic protein perforin (Prf1) that lead to reduced perforin expression (and reduced viral immunity), associate with MS development only in men (105). In addition, infection of mice with Theiler’s murine encephalomyelitis virus (TMEV) leads to increased development of the autoimmune demyelination in male compared to female mice as a result of weaker T cell and antibody responses and reduced viral clearance in the males (106, 107).

In addition, there is evidence from human studies that the repertoire of CD8^+^ T cells, particularly those specific for herpes viruses that latently infect humans including EBV and cytomegalovirus (CMV) (a virus that is not implicated in MS) undergoes shifts from a naïve towards an effector memory or senescent status with aging (108, 109). It is thought that this shift is caused by the reactivation of these viruses that leads to a selective expansion of viral-specific T cell clones and a loss in the diversity of the TCR repertoire (108). Interestingly, in the case of CMV, the shift in T cells from a naïve to more senescent status with aging is more striking in men and is associated with higher expression of TNF (110), a cytokine that is toxic to both OLs and neurons (111, 112). Coinciding with this, a greater reduction in the diversity of the CD8^+^ T cell repertoire is also described for men compared to women with MS (113) and MS men have a larger fraction of CD3^+^ T cells that produce TNF compared to female counterparts (114). A shift from a naïve and towards a T effector status has been also observed for EBV-specific CD8^+^ T cells in healthy individuals with natural aging (109). However, it remains to be seen whether this occurs differently between men and women and whether EBV latent infection is driving the greater loss in TCR diversification and acquisition of higher TNF expression seen in male CD8^+^ T cells in MS.

In conclusion, CD8^+^ T cell immunity is overall more robust females, which may impact autoimmune initiation by modulating the efficiency of viral clearance and tissue damage. In addition, there is a greater perturbation of the CD8^+^ T cell repertoire with aging in males with MS that correlates with increased TNF production.

## 5 Sex differences in B cells and anti-EBV humoral responses

Immune infiltrates in the perivascular spaces contain B cells (115). B cells also are the major cell type that comprises the ectopic follicle-like structures detected in the meninges in half of SP-MS patients (116). The appearance of these follicle-like structures associates with myelin and neuronal damage in the underlying cortex (subpial cortical lesions) (117–120) and associates with the development of a more rapid and severe course of MS (117). Though cortical lesions have been reported to be more prevalent in males compared to females with MS either upon autopsy (49) or by MRI in living patients (54), these studies lumped cortical, leukocortical, intracortical, and subpial lesions together, making it unclear whether the subpial lesions were more abundant in males. This distinction is important since leukocortical and intracortical lesions resemble WM lesions in that they are driven by intravascular inflammation whereas subpial lesions associate with meningeal inflammation (121). Magliozzi and colleagues did note a trend for a higher female to male-ratio in the MS brain specimens that contained B-cell-follicles compared to those that did not, suggesting a tendency for a higher probability of women to this immune reaction in MS (120). Since B cells and plasma cells in the meninges are expected to be the major producers of intrathecal Ig, IgG index and the presence of oligoclonal bands have been evaluated in the cerebral spinal fluid of male and female MS patients, but were found to not differ between the sexes (122). However, larger cohort studies did report that oligoclonal band negative patients with MS were more likely to be male (123, 124). These data suggest a tendency for an increased presence of B cell clusters and oligoclonal bands in females, which is consistent with the knowledge that healthy females have higher numbers of immunoglobulin (Ig) M-producing memory B cells in peripheral blood (125, 126) and exhibit overall better antibody responses to vaccination (126) than males.

EBV infection is a risk factor for MS and B cells are the cellular reservoir for this virus (101). Some, but not all (127, 128) studies, have reported detecting EBV-infected B cells and plasma cells in the meningeal ectopic follicle-like structures (129–131) and in perivascular spaces within the cortex (132) in MS brains. It has been speculated that EBV infection can lead to the over-activation of B cells, including those in the meningeal ectopic follicle-like structures [reviewed in (133)]. Humoral responses against the EBV encoded transcription factor Epstein-Barr virus nuclear antigen 1 (EBNA-1) precede conversion to clinically-definite MS (134, 135). Those with clinical isolated syndrome or MS having the highest quartile of serum IgG antibodies specific either for the viral capsid antigen (VCA) in EBV or EBNA-1 have been reported to have greater T2 lesion burden and extent of whole brain or GM atrophy compared to patients having lower IgG levels (136–138). Despite this link between the EBV and MS progression, studies that examined sex differences in B cell responses against EBV are scarce. Healthy human females are reported to have higher titers of anti-EBV antibodies (139–141) and are more likely to be seropositive (140) than healthy males. One study of Kuwaiti MS patients, reported that serum titres of anti-EBNA1 and anti-VCA were higher in males with MS compared to healthy male controls; levels did not differ between MS males, healthy females and MS females (142). The selective increase in EBV antibodies in males with MS could indicate greater re-activation of the virus in this sex.

The success of anti-CD20 B cell-depleting therapy in both RR-MS and in PP- MS (143, 144) has highlighted the importance of B cells in MS disease mechanisms. These therapies deplete memory B cells in the blood, but do not impact intrathecal IgG levels (145). This B cell depletion is also associated with a decrease in T effector and terminally-differentiated T cell populations (146), which has been speculated to occur as a result of reduced B cell antigen presenting function (147) or depletion of a highly-activated CD20^+^ subset of T cells that acquire CD20 from B cells through interactions (148). With respect to sex, anti-CD20 therapy is an effective treatment in both men and women with MS (149). To the best of our knowledge, there exist no reports that have evaluated sex differences in the antigen presenting function of B cells in MS before or after anti-CD20 therapy. Therefore, at present there are no strong data to suggest a role for B cells in driving sex differences in MS progression.

## 6 CD56^bright^ NK cells are increased in the peripheral blood of females with MS

Human NK cells can be subdivided into two major subsets according to the expressions of CD16 and CD56: the CD16^+^CD56^dim^ subset and the CD16^-^CD56^bright^ subset. Both of these subsets are cytolytic, however, the CD56^dim^ subset is the more mature of the two and is capable of killing target cells without prior sensitization, whereas the CD56^bright^ subset is considered to have a regulatory function (can lyse activated T cells), expresses higher amounts of modulatory cytokines, and acquires cytolytic function only after prolonged activation (150). The CD56^dim^ NK cells are the major population found in blood, whereas the CD56^bright^ subset is more prominent in secondary lymphoid organs (150) and in the CSF and perivascular spaces in MS lesions (151). Whether there exist sex differences in the abundance or activity of NK cells within MS lesions or in the CSF has not been investigated; however, one study has investigated sex differences in the levels of NK cell subsets in peripheral blood of MS patients and healthy individuals. Though no sex differences were detected in the frequency of NK subsets when comparing healthy controls or MS patients, CD56^dim^ NK cells were found to be more activated in the blood of males with MS compared to healthy males, whereas the CD56^bright^ NK cells were increased in the blood of females with MS relative to healthy females, suggesting increased NK regulatory function in this sex (152). Future studies should examine the proportion and functionality of these subsets in MS lesions or the cerebral spinal fluid to gain further insights into sex differences in the regulatory function of CD56^bright^ NK cells in this disease.

## 7 Female microglia become more reactive with age compared to male microglia

As discussed previously, men have a higher predominance of smoldering lesions with active rims of microglia compared to women (49, 50). Consistent with this, one study that evaluated the expression of pro-inflammatory cytokines (IL-1β, IL-6, and TNF) in MS lesion samples, reported that compared to female lesions, male lesions express higher levels of *TNF*, a factor that is expressed by microglia (153).

In adult mice, microglia density is reported to be higher in males than in female in the cortex, hippocampus, and the cerebellum (154). Although the basal phagocytic activity of microglia does not differ between males and female adult mice *in situ*, male microglia in the cortex have a rounder morphology and intracellular recordings of membrane current responses in individual microglia after application of ATP suggest increased expression of the P2X receptor (that senses of ATP released from damaged cells) in male compared to female microglia in the cortex (154). Cortical microglia from males also express higher levels of MHC Class I and II *in situ* compared to the females (154). However, it still remains unclear whether these altered microglia characteristics are due to factors in the environment of the microglia (e.g., more T cells or cell damage) or sex differences in the potential of microglia to respond to their environment.

In regard to the latter, a number of studies have characterized sex differences in microglia phenotype in rodents *ex vivo*. For example, marked sex differences are detected in microglial gene expression after microglia cells are isolated from mice and cultured *in vitro* or passaged (an *in vitro* model of microglia senescence). Young female microglia express higher levels of pro-inflammatory genes (such as IL-6, TNF, Toll-like receptor 4 [TLR4]) compared to male microglia after stimulation with IFN-γ; however, when the microglia are passaged, female microglia lose this ability to upregulate these pro-inflammatory factors, whereas male microglia uniquely upregulate IL-1 mRNA in the absence of IFN stimulation (155). In addition, female aged microglia exhibit an increased ability to phagocytose neuronal debris *in vitro* compared to male aged microglia (155), which may aid in myelin repair. Though this culture system is artificial, it raises the possibility that sex differences in microglia senescence could be a factor in sex differences in MS progression.

Microglia gene expression has been also studied *in situ* or after direct isolation from young adult male and female mice. A study by Villa et al. that compared gene expression profiles in male and female microglia reported that the male microglia express higher levels of genes involved in inflammatory processes compared to female microglia; male enriched genes contained binding sites for NF-κB and RUNX1 transcription factors (156). By contrast, female microglia expressed higher levels of genes associated with morphogenesis, development, and cytoskeletal organization (156). Interestingly, these sex-dependent patterns of gene expression were not sensitive to estrogen treatment and were maintained even after *in vitro* culture or transplant into mice of the opposite sex (156), suggesting that they were specified earlier in development by either sex chromosome complement or early life hormonal surges. Similar to these findings, Hanamsagar and colleagues reported that male microglia are more reactive to lipopolysaccharide (LPS) or bacterial stimulation *in vitro* and that this sex difference was apparent up to day 60 of post-natal development (157). A study by Guneykaya and colleagues that examined microglia in 13 week-old mice reported that MHC Class I and II were expressed at higher levels by male compared to female mice in the cortex suggesting increased antigen presenting cell capacity of the male microglia (154). Analysis of gene expression in the isolated microglia revealed greater expression of genes involved in the defense to bacteria and greater protein expression of TLR and S100 proteins. In addition, male microglia exhibited higher expression of genes involved in “ATP binding” and higher protein expression of the ATP-sensing purinoceptors P2X 4 and 7 and P2Y purinoceptor 12 (154). On the other hand, female microglia were enriched for transcripts involved in “GABA and glutamate receptor activity”, ubiquitin protein activity”, and “magnesium transport” and showed higher protein expression of interferon regulatory factor-3 (Irf-3), which is a factor that is involved in inducing Type I IFN (154). Consistent with the latter, Thion et al. reported that female murine microglia have higher expression of genes involved in type I IFN signalling (158). This study also noted that the animal facility where the mice are housed (microbiota/diet) had a large impact on microglia gene expression (158), providing a possible explanation for the disparities in microglial signatures reported by different groups. Altogether, these findings suggest that male microglia may be more responsive to bacterial products or ATP released from dying cells, whereas female microglia may exhibit heightened IFN production and responsiveness.

Studies that evaluated murine microglia in the context of aging have suggested that female microglia acquire a more pro-inflammatory phenotype in the steady state and during disease. A study that measured gene expression in the hippocampus of young adult, middle-aged, and old male and female mice concluded that pathways related to inflammation, macrophage activation, and activation of the “sensosome” (i.e., TREM2, complement expression) in microglia were increased with middle- and old-age in female mice. This increase occurred, but was less striking, in the males (159). Comparison of these aging-related transcripts against gene expression profiles of CNS resident cell types suggested that the age-related genes were enriched for microglia-specific transcripts (159). Similar to these findings in mice, a study of aging in the human brain reported finding more extensive upregulation of inflammatory genes (e.g., TLRs, complement CD3, CD14) and pathways (antigen presentation, IFN-regulated, macrophage activation) in female compared to males. Furthermore, a study that evaluated microglia in a genetic model of Alzheimer’s disease reported that female microglia experience a greater downregulation of genes associated with a homeostatic phenotype and a greater upregulation of genes associated with a damage-associated microglia phenotype with age (160). Striking sex differences were also seen in the morphology and metabolism of the microglia in that the male microglia were more amoeboid and less glycolytic than the female microglia (160). Another study that evaluated microglia activation in aged wild type mice, and genetic mouse models of Aβ and tau pathologies, using 18-kDA translocator protein positron emission-tomography (TSPO-PET) reported detecting a higher signal for female microglia in both the wildtype and Aβ mice, but not in the tau model of Alzheimer’s disease (161). This TSPO-PET signal correlated with increased staining for microglia activation markers Iba1 and CD68 in the female brain sections. Similarly, TSPO-PET studies in humans describe a higher TPSO-PET signal in the brains of healthy women versus healthy men (162).

It has been difficult in EAE studies to interpret whether there are sex differences in microglia phenotype, since T cells infiltrate the CNS to a greater extent in the female than male mouse [reviewed in (5)]. Nonetheless, EAE studies identified that p38α signalling operates in a sex- and cell-dependent manner to modulate CNS inflammation in this disease. When the activity of this kinase is knocked out in the peripheral myeloid compartment, EAE is initially more severe in both sexes (163–165), but then becomes progressive only in mice of the female sex (164). In contrast, when p38α expression is knocked down only in microglia, EAE is exacerbated more so in the males (165). Deficiency of p38α in male microglia associates with a pronounced upregulation of a number of pro-inflammatory genes; these same genes were not altered or were downregulated in the female microglia with p38α deficiency (165). Thus, there exist sex differences in the activity of a key signalling intracellular signalling pathway in microglia.

In conclusion, the literature suggests that female microglia are more reactive to IFNs and become pro-inflammatory with age, which contrasts with the finding that men with MS exhibit more smoldering microglia activation at rim of WM lesions during disease progression. It is possible, that the increased microglia seen in male lesions is to an increased responsiveness to damage-associated signals such as ATP, the increased presence of inflammatory mediators secreted by lymphocytes in the perivascular spaces, or enhanced iron release from OLs (discussed in Section 9).

## 8 Astrocytes may be more reactive in males with EAE and MS

Astrocyte activation also accompanies microglia activation in WM lesions in MS and EAE (166–168). In MS, the disruption of astrocytic end-feet around blood vessels and the appearance of hypertrophic astrocytes at the rim of WM lesions is one of the earliest histopathological features in the acute MS lesion, and astrogliosis, defined as increased expression of glial fibrillary acidic protein (GFAP), occurs alongside chronic demyelination and axon loss in the core of demyelinated lesions (169). Astrocytes become reactive upon exposure to pro-inflammatory mediators and danger signals in EAE and MS lesions (170). Studies in EAE have shown that astrocytes are also key producers of CCL2, which mediates immune cell recruitment to the CNS (171). In addition, astrocytes can acquire a neurotoxic phenotype in MS and EAE (172). Furthermore, protein products that are released from dying astrocytes such as GFAP and chitinase-3-like protein 1 (C3L1) are detected at increased levels in the CSF and serum of MS and are prognostic biomarkers for disease progression (173–175).

Astrocytes, like microglia, can also exert anti-inflammatory and neuroprotective activities in MS and EAE [reviewed in (170)]. Astrocyte loss-of-function experiments in mice have demonstrated that astrocytes, overall, have a protective function in EAE (176, 177). The end-feet of these cells forms the *glia limitans* of the blood brain barrier (170). In addition, these cells directly connect with OLs through gap junctions and contact many neurons where they produce energy, neurotransmitters, and buffer ions to support the health and conduction of neurons and OLs (170). Astrocytes also produce low levels of anti-inflammatory cytokines such as IL-10 and are important producers of brain-derived neurotrophic factor (BDNF) (170), which is protective against axon injury in EAE (178) and cuprizone-induced demyelination (179).

Regarding sex differences in astrocytes, levels of GFAP and C3L1 levels are not reported to differ between male and female MS patients in the CSF (65, 174). However, another astrocyte-produced factor, macrophage inhibitory factor (MIF), has been implicated to be a male-specific factor in predicting MS progression. MIF is highly expressed by hypertrophic astrocytes in chronic MS lesions (180) and the levels of MIF in the serum correlate with EDSS score and the number of black holes (areas of permanent neuronal loss) on MRI (181). Males with progressive forms of MS also exhibit higher levels of MIF in the CSF compared to females (180, 182). Furthermore, polymorphisms in the *MIF* gene that associate with increased MIF expression predict the development of a progressive MS course only in males (182). Studies in the EAE model have confirmed that MIF has pro-inflammatory activities during neuroinflammation (183) and that this factor is expressed at higher levels in the spinal cords of male compared with female mice during disease (184).

Besides these findings for MIF, it remains controversial whether there exist sex differences in astrocyte phenotype in EAE, with some studies showing greater activation in males and others showing greater activation in females. For example, post-natal isolated astrocytes derived from males have been shown to express higher levels of IL-1, TNF, and IL-6 mRNA after culture in the presence of LPS compared to female astrocytes (185), suggesting that there may be intrinsic sex differences in the potential of astrocytes to become activated in response to bacterial stimuli. Studies in a murine model of stroke also reported that male mice exhibit worse outcomes, correlating with more extensive astrocytic changes in calcium flux, release of S100β protein, and altered polarization of expression of aquaporin 4 (expressed in astrocyte end-feet); this occurred despite females having increased microglia activity (186). More extensive astrocyte and microglial activation has also been observed in males in a cortical brain injury model (187). In this case, males showed a higher density of microglia at the edge of the wound that correlated with higher expression of CCL2 by astrocytes (187). In the cuprizone-induced demyelination/remyelination model, male mice exhibited greater deficits in conductivity in the corpus callosum (CC) in the remyelinating phase of disease that associates with higher GFAP and Iba1 staining (188). In MOG peptide 35-55 (MOG p35-55)-induced EAE in C57BL6/J mice, some studies report that males exhibit more pronounced GFAP immunoreactivity in the spinal cord (189, 190); however, this is not observed in all studies: one study found equivalent GFAP expression in the spinal cord of males and females and that it was the females that had a higher number of C3^+^ GFAP^+^ cells (marker of neurotoxic astrocytes) in the optic nerve (172). Taken together, these studies suggest that males may be more prone to astrogliosis in some models of brain injury.

Astrocytes also are key mediators of the neuroprotective effects of gonadal hormones in EAE and MS. Mice that have deletion of estrogen receptor (ER)-α specifically in astrocytes, but not in neurons, are resistant to the neuroprotective responses to estrogens (191). Further, treatment with an ER-α ligand substantially ameliorates clinical symptoms, peripheral immune cell infiltration, and axonal loss in female mice with EAE; effects are exclusively mediated through astrocyte-induced ER-α (191). Further, treatment with an ER-α ligand reduces the expression of chemokines CCL2 and CCL7 by astrocytes, suggesting a role for estrogens in regulating the severity of developed inflammation (72). However, a drawback of these studies is that they were only conducted in female mice, thereby not permitting an evaluation of how this biology factors in one sex versus another.

Consistent with hormone signalling being protective in astrocytes, a histopathological study of female and male MS lesions detected an upregulation of sex hormone receptors in MS lesions compared to the surrounding healthy tissue and NAWM and found that this occurred in a sex-dependent manner (153). Females preferentially upregulated the progesterone receptor and an enzyme involved in progesterone synthesis, whereas males upregulated TNF, ER-β and aromatase, which can convert testosterone to estradiol (153). Immunolocalization studies confirmed that the expression of the hormone receptors and hormone synthesizing enzymes in the GFAP+ astrocytes. Though associative, these findings suggest that progesterone and estrogen are part of an endogenous protective mechanism in MS and that females harness progesterone to mediate this protection, whereas males harness ER signalling, which may be less effective in inhibiting inflammation as evidenced by the higher expression of TNF (153).

Taken together, these finding suggest that astrocytes produce and respond to steroids and mediate the neuroprotective effects of ovarian hormones in the brain. There is evidence from animal studies that male rodents may be more prone to develop reactive gliosis during injury and neuroinflammation and that MIF-1, a factor that produced by astrocytes in MS lesions associates with disease progression in males with MS.

## 9 Iron accumulates at the rim of active lesions and in deep GM nuclei more so in males with MS

Iron is present in the healthy brain where it is essential to supporting the normal metabolic functioning of neurons and glia and myelin repair mechanisms (192). Iron is primarily stored in the non-toxic ferric (Fe^3+^) form bound to ferritin and these stores are contained in OLs and to a lesser extent in neurons, microglia and astrocytes (193, 194). However, during acute MS attacks iron is liberated from dying OLs where it collects in granules in the perivascular space and can be taken up by microglia and macrophages (194). During this process, there is a conversion of ferric iron into the more reactive ferrous (Fe^2+^) form, which can interact with oxidants produced by microglia and macrophages to generate the highly reactive and neurotoxic hydroxyl molecule (192). Ferrous iron is localized in areas of axon injury and oxidative damage in acute WM lesions and in deep grey matter nuclei in MS (194), suggesting that it contributes to the development of neuronal injury in this disease. Over time, this pathological release of iron from OLs leads to a depletion in iron in demyelinated cores of WM lesions and in the NAWM and to an increase in iron in microglia at the rim of smoldering lesions as well as the deep grey matter nuclei. Iron-laden microglia acquire dystrophic features such as fragmented processes, suggesting that these cells are damaged by increased iron load (194).

Regarding sex differences, QSM MRI studies in living MS patients have demonstrated that iron accumulates more so in men compared to women both at the edge of smoldering WM lesions (52) and in the caudate and putamen (195), which are highly susceptible to neurodegeneration and atrophy in MS (192). However, a pathological study of iron in deep GM nuclei reported finding no sex differences in the extent of demyelination, and if anything female lesions showed a higher iron density and staining for oxidative lipids (193), suggesting that the interaction of iron with ROS to produce oxidant species may be higher in females. On the other hand, iron load is higher in men as compared to women in deep grey matter nuclei during healthy aging (196–198) and in MS (195) suggesting that there is more iron in these regions that can be liberated from male OLs if they are damaged.

One study that investigated the effects of iron overload on the development of EAE in Dark Agouti rats reported that iron treatment accelerated development of EAE in females, but resulted in greater progression and higher mortality from disease in the males (199). This more extensive damage in the males correlated with a higher degree of CNS lipid peroxidation and demyelination and gliosis in the spinal cord, providing proof of concept that high iron can accelerate neurological progression (199). Taken together, the finding of a greater accumulation of iron in the brains of males in areas that are implicated in MS progression is compelling, but further studies are necessary to define whether iron is a pathogenic factor in the increased microglia activation and neurodegeneration seen in WM lesions the males or is an epiphenomenon related to the increased microglia activation or OL damage at these sites.

## 10 Sex differences in susceptibility to demyelination and repair

It has been suggested that females with MS have an advantage when it comes to myelin repair in lesions (55). Studies in rodents have described a number of sex differences in myelin biology in the steady state and in toxin-induced demyelination/remyelination models that may account for this sex difference. For example, a study that evaluated myelination in different brain regions in rodents found that the density of OLs was higher in the CC, the fornix and the spinal cord of males versus females (200). This was seen in a variety of rodent strains and was accompanied by higher expression of mature myelin proteins such as proteolipid protein and myelin basic protein in the males (200). On the other hand, it was observed that female OLs turned over at a faster rate than the male OLs (200). Castration of the males reduced the number of OLs and increased the generation of new OPCs in the males (200), suggesting that sex differences in myelination are regulated by male gonadal hormones. Similar sex differences in myelination were observed in the CC of mice in another study that also provided evidence of a role for the post-natal surge in androgens (i.e., mini-puberty) in mediating these sex differences (201). Consistent with these findings, a study that compared the *in vitro* proliferation and differentiation of OPCs that had been isolated from perinatal (1-2d old) rats noted the same phenotype of increased proliferation of the female OPCs and a greater ability of the male OPCs to differentiate to mOLs *in vitro* (202). Besides these sex differences in myelin turnover and differentiation, female OPCs exhibit more rapid migration compared to male OPCs by scratch assay, and are less vulnerable to *in vitro* glucose deprivation compared to male OPCs (202).

Sex differences in the extent of demyelination and/or remyelination have been observed in rodent models of toxin-induced demyelination. For example, a study of cuprizone-induced demyelination in SJL mice reported that there is a greater sparing of mature OLs in the CC of females compared to males in the demyelinating phase of disease (203). This sex difference was not accompanied by differences in the extent of microglia or astrocyte activation or in the density of OPCs between males and females (203). By contrast, no sex differences in MBP staining were seen in the demyelination or remyelination phase in a cuprizone-induced demyelinating model in C57BL/6 mice (188, 204). However, an experiment in C57BL6/J mice that evaluated the effect of gonadectomy on the kinetics of demyelination and remyelination revealed that castration and ovariectomy hindered remyelination after cuprizone withdrawal (188). This defect in remyelination with gonadectomy was restored by exogenous treatment with estradiol and testosterone, which can be aromatized to estradiol, but not dihydrotestosterone (DHT), which binds the androgen receptor, but cannot be aromatized to estradiol; this effect of sex hormones correlated with an increased number of OLs in the CC (188). These findings suggest that estradiol is a factor that can promote remyelination in both males and females (188).

Further supporting myelin-protective effects of estradiol, treatment with pregnancy levels of estradiol prevents the loss of OLs in the CC during the demyelinating phase in the cuprizone-induced demyelination model that correlates with a delay in microglia recruitment and activation (205). Estrogen treatment also improves remyelination in a more chronic (6 or 9 weeks) cuprizone feeding regimen and this correlated with an increase in the number of OPCs, immature and mature OLs in the CC (206). These protective effects are negated in mice that were deficient in ER-β in cells of the OL lineage (206). Further studies using a specific ER-β agonist revealed pro-myelinating effects of this agonist in EAE that associated with an upregulation of the expression of genes related to cholesterol synthesis in the OPCs (206). In addition to estradiol, the ovarian hormone progesterone has been shown to stimulate the proliferation, migration and maturation of OPCs *via* the insulin-like growth factor pathway *in vitro* (207) and the additive activities of estradiol and progesterone can protect against demyelination in the CC during the demyelinating phase of the cuprizone model (208). Thus, estradiol at physiological concentrations can promote remyelination and when administered at pregnancy levels along with progesterone can also spare myelin in toxin-induced demyelinating models.

The role of sex chromosome complement on demyelination and remyelination has been also investigated in the cuprizone demyelinating/remyelination model using the four core genotype mice. When these four sets of mice (gonadal XX females, gonadal XX males that are transgenic for the testes determining gene Sry, gonadal XY males, gonadal XY-Sry^-/-^ females) are gonadectomized and subjected to cuprizone feeding followed by a period of cuprizone withdrawal, it was found that mice that had an XX chromosome complement had significantly higher electrical conductivity in the CC during the remyelinating phase of disease. This greater recovery correlated with higher proliferation of the female OPCs compared to the male OPCs (209).

Aging may also be a factor in the sex differences in myelination. In the ethidium-bromide-induced demyelination model, ethidium bromide is injected into the caudal cerebellar peduncle, which results in focal demyelination and this is followed by a period of spontaneous remyelination over 4 weeks. When this experiment was conducted in young rats, no sex differences were seen in myelination (210). However, in older rats, which remyelinate more slowly, older females showed more extensive myelination at 8 weeks post injection of ethidium bromide compared to males (210). Gonadectomy did not influence these sex differences, suggesting that they were hard-wired in OPCs at an earlier stage of development.

Neuronal progenitor cells (NPCs) are multipotent cells that can self-renew, and migrate to injured WM regions in both EAE (211) and in cuprizone-induced demyelination model (212) where they can give rise to astrocytes, OPCs, and neurons. These cells are located in the subventricular zone and the dorsal lateral horn in the brain. A study that examined sex differences in the proliferation of NPCs in the steady state in C57BL6/J mice, found no differences in the proliferation of these cells in young adult male and female mice; however, with middle age, the proliferation of NPCs declined more so in the males and this sex difference was reversed by castrating the males. OPCs numbers in the CC changed in lock step with the number of BrdU^+^ NPCs in the dorsal lateral horn suggesting that the progenitor cells were differentiating into OLs in the CC. However, no sex differences were observed in the number of proliferating NPCs in other mouse strains. Thus, while results vary between rodent strains and model systems, the data collectively point to females having an advantage when it comes to either sparing or regenerating myelin, particularly during aging.

## 11 Sex differences in neuron vulnerability and circulating levels of neurotoxic molecules

In addition to the mechanisms described above, there is evidence from both murine EAE and *in vitro* studies that male neurons are more vulnerable to the pro-inflammatory molecules and ROS and RNS generated during neuroinflammation. For example, studies in EAE that utilized the four core genotype mouse model provided strong evidence for a role for XX chromosome complement in protecting neurons from damage (213). Studies were performed where gonadal female mice (XX or XY-*Sry^-^*^/-^) were irradiated (which removes the immune system) and then provided with either XX and XY-*Sry^-^*^/-^ bone marrow, thereby generating mice that had ovaries and equivalent hormone levels but had either an XX or XY-*Sry^-^*^/-^ genotype in the radioresistant (microglia, neurons, astrocytes) or radiosensitive (immune system) compartment. When the radiation bone marrow chimeras were induced to have EAE, XY-*Sry^-^*^/-^ gonadal female recipients developed more severe clinical symptoms than XX gonadal females regardless of chromosomal sex in the immune system (213). The more severe EAE in the XY-*Sry^-^*^/-^ females did not correlate with the extent of T cell or macrophage infiltration in the CNS, but instead with more severe loss of myelinated axons and neurons (213). Interestingly, the major correlate of neurodegeneration in XY-*Sry^-^*^/-^ females was higher expression off the X chromosome-encoded gene *Tlr7* in neurons (213). Additional work revealed that this was due to lower methylation of a gene cluster that included *Tlr7* on the inherited maternal versus paternal X chromosomes: males in only inheriting the maternal X had higher expression off this gene cluster compared to the females which had a mosaic of expression of these genes off the paternal and maternal X chromosome (214). Thus having an XY chromosome complement made male neurons more vulnerable to inflammatory insults.

Consistent with this, *in vitro* studies of neurons have also revealed cell-intrinsic differences in the viability of male and female neurons. For example, when primary hippocampal neurons are harvested from embryonic day 16-17 rat pups and cultured with a variety of toxic agents, XY neurons are more vulnerable to oxidative, nitrosative stress, and glutamate excitotoxicity compared to XX neurons (215). Treatment with N-acetylcysteine, which raises glutathione levels, reverses these sex differences pointing to sex differences in antioxidant defence mechanisms in neurons (215). This study also demonstrated that XX and XY neurons proceed through different pathways of apoptosis during nitrosative stress, with the XY neurons utilizing an apoptosis-inducing-factor-dependent pathway and XX neurons utilizing a cytochrome C-dependent pathway (215). Together, these studies further point to intrinsic sex differences in the neuron vulnerability driven by sex chromosome complement.

One reason neurons die in MS is because mitochondria become damaged as a result of oxidative and nitrosative stress, leading to respiratory chain deficiencies, energy deficits, and increased mitochondrial ROS production (80). Studies in rodents have provided strong evidence that mitochondria from female brains or neurons have greater respiratory function and higher resistance to oxidative damage in response to treatment with neurotoxins. For example, mitochondria isolated from brains of young female mice exhibit higher NADH-dependent respiratory rate compared to male microglia, that associates with a higher activity of pyruvate dehydrogenase and increased glutathione concentrations in the female mitochondria (216). This sex difference correlates with brain progesterone levels and is negated by aging or ovariectomy of the females, but not by castration of the males (216). Consistent with this, a study in rats showed that mitochondria that were isolated from the brain exhibited a reduction in function with aging, especially in the males who showed reduced mitochondrial capacity compared to the females (217). In addition, *in vivo* treatment of female rats with the ovarian hormones E2 and progesterone increased the respiratory rates of brain mitochondria, which correlated with increased cytochrome C oxidase and reduced rate of oxygen leak and lipid peroxidation (a sign of oxidative damage) in the mitochondria (218). Studies in a murine model of Alzheimer’s disease have also shown that female neurons are more viable than male neurons due to the female mitochondria being less vulnerable to calcium overload (219).

Sex differences in neuronal vulnerability linked with altered mitochondrial function have also been seen in studies that treated mice or neurons with neurotoxins that target the respiratory chain including 1-methyl-4-phenyl-1, 2, 3, 6 (MPTP) and β-N-oxalyl amino-L-alanine (L-BOAA). For example, treatment of mice with MPTP results in a loss of dopaminergic neurons in the substantia nigra only in male mice, which correlates with lower levels of glutathione mRNA in the male brain; females become sensitized to the neurotoxin when treated with an ER-α antagonist (220). Similarly, when mesencephalic neurons are cultured *in vitro* with another toxin, 6-hydroxydopamine, which is used to induce experimental Parkinson’s disease, female neurons are less vulnerable to apoptotic and necrotic death and this correlates with increased expression/activity of mitochondrial respiratory subunits and reduced ROS production (221). In addition, treatment of mice with L-BOAA, which induces mitochondrial protein oxidation, only elicits motoneuron death in male mice and this protection in females associated with higher expression of glutaredoxin, which is a component in the glutathione antioxidant system in the CNS (222). Females are made vulnerable to the neurotoxin by ovariectomy or by knocking down glutaredoxin (222).

Homocysteine is an amino acid generated from the dietary methionine that has toxic activities on neurons even when present at physiological concentrations (~10 µM) [reviewed in (223)]. Studies have demonstrated that *in vitro* treatment of neurons with homocysteine contributes to increased mitochondrial dysfunction and oxidative stress, and stimulates apoptotic and excitotoxic pathways in these cells (223). Accordingly, a number of studies have reported that homocysteine is present at significantly higher levels, close to the range of neurotoxicity, in the plasma of male compared to female MS patients (224, 225). One study that investigated the concentrations of homocysteine in the plasma in MS patients and healthy controls reported it to be uniquely elevated in the male MS group (224). Another study reported homocysteine levels to be higher in the males compared to the females both in healthy individuals and those with MS (225). This study further showed that homocysteine levels associated with more rapid progression, increased disability, and the development of a more progressive course of disease (225). Therefore, elevated homocysteine levels could be an additional factor that is contributing to increased neurodegeneration in males.

Taken together, studies in rodent models suggest that XY neurons are more vulnerable to inflammatory and oxidative stresses compared to XX neurons due to sex differences in X chromosome methylation and effects of ovarian hormones in promoting mitochondrial respiration and antioxidant defense mechanisms. Further homocysteine is a neurotoxic factor that circulates at higher levels in males. If these findings translate to MS, it would give female neurons a survival advantage in the pro-inflammatory environment of the MS lesion.

## 12 Sex differences in gut microbiota

Gut dysbiosis, which is defined by a reduced diversity or altered composition of bacterial species in the gut, has been reported to occur in MS [reviewed in (226)]. Besides having a potential to cross-activate autoreactive T cells through antigen molecular mimicry (227), gut bacteria can promote or modulate systemic inflammation through bacterial translocation, by increasing gut permeability, or through differential production of bacterial metabolites (228). Proof of concept that the microbiome can modulate the immune system in MS was provided by the seminal study that colonized germ-free mice with the microbiota from twins that were discordant for MS (229). Though sequencing of the microbiota did not identify major differences between the MS or healthy microbiota, the microbiota from the RR-MS patient had the effect of increasing the incidence of spontaneous EAE in recipient TCR transgenic mice compared to the healthy twin microbiota and this associated with a reduction in IL-10 producing T regulatory cells (229). Furthermore, there is strong evidence that production of metabolites by certain species of gut bacteria is protective in MS. For example, short-chain fatty acids (SCFA) butyrate and propionate protect against the development of EAE (230) and MS autoimmunity (231) by shifting the balance between T effector cells and T regulatory cells. Indeed, a clinical trial in MS showed that propionate treatment reduced relapses, stabilized disability, and reduced brain atrophy after 3 years, providing proof of concept that SCFAs can regulate disability progression (231). Studies in mice have provided strong evidence that the bacterial composition of the gut and lung microbiota and generated metabolites can affect the phenotype of microglia and other glial cells in the CNS (232–235).

Though a large majority of studies of the microbiota in MS have focused on RR-MS, several studies have reported on microbiota changes in progressive MS (236–239). Although differences were found in the composition of gut bacteria between healthy people and MS patients, no major differences were found between SP-MS and RR-MS patients; with the exception of a small number of bacterial species (238, 239). For example, a study in Japan that sequenced gut bacteria in people with various MS subtypes found increased expression *Streptococcus* and *S. parasanguinis* in SP-MS compared to RR-MS (238). Metagenomic analysis revealed an increased presence of microbial genes involved in DNA mismatch repair and reduced microbial carbohydrate metabolism, whereas metabolite analysis revealed an increased ratio of cysteine persulfide to cysteine (238). The authors interpreted these data to indicate that there is excessive DNA oxidation in the gut of SP-MS patients and speculated that it was due to the overgrowth of *Streptococcus* that can produce hydrogen peroxide. Unfortunately, this study was small and the authors did not disaggregate their data according to sex.

A study of the CLIMB cohort in Boston reported finding 16 species of bacteria that were differentially abundant between RR-MS and SP-MS patients including *Bacteroides*, *Enterobacteriaceae* and *Clostridium g24 FCEY* (237). They also found that the abundance of the *Clostridium* species associated with higher EDSS and fatigue scores in the patients, suggesting that these bacteria could be a driver of disease progression (237). Though this study identified sex to be a contributor to microbiome variation, the authors controlled for this variable as opposed to presenting data by sex, making it difficult to study the interaction of sex with microbiota composition in disease.

Sex differences in gut microbiota have been described for both healthy humans and rodents [reviewed in (240)]. *Akkermansia*, and its family, *Verrucomicrobiaceae*, and *Bacteroides* spp. are more abundant in healthy female mice (241, 242) and female humans (243, 244) compared to male counterparts. Although the genus *Akkermansia* is increased in progressive MS and EAE (237, 245), its presence correlates with lower disability in both progressive MS (237) and in the progressive phase of EAE in NOD mice (246). The gut microbiota of animals with RR-EAE has more *Bacteroides* spp. compared with mice with chronic-progressive EAE (245). On the other hand, SCFA-producers, whose abundance affects Treg/Th17 balance (231) and negatively correlates with disease activity in PP-MS (236), are more abundant in the gut microbiota of pre-menopausal females than in males, but not in post-menopausal females (247). If this sex difference is also preserved in MS, it may explain the more profound brain atrophy, axonal damage and physical and cognitive deficits seen in men and disability worsening reported for post-menopausal females in some studies (248–250) (also see Section 2).

Importantly, sex hormones can drive sex differences in gut microbiota (251) and vice-versa (252). Thus, amelioration of EAE by 17β-estradiol is associated with an increase of *Lachnospiraceae* and a decrease of *Erysipelotrichaceae* in gut microbiota (253). These bacterial families associate with MS progression: while *Erysipelotrichaceae* correlates with brain atrophy in progressive MS, and *Lachnospiraceae* negatively correlates with fatigue, anxiety, and depression (237). *Akkermansia* abundance is reduced when female mice are treated with androgens (254), and castration switches the gut microbiota of male NOD mice to a more feminine one, abrogating male protection against type 1 diabetes (255).

Altogether, sex differences in gut bacteria have been described for healthy mice and humans, and the bidirectional relationship described between sex hormones and gut microbiota suggests that microbiota changes have a potential to drive sex differences in both peripheral and CNS populations that are important to MS progression.

## 13 Summary

**Figure 1:**
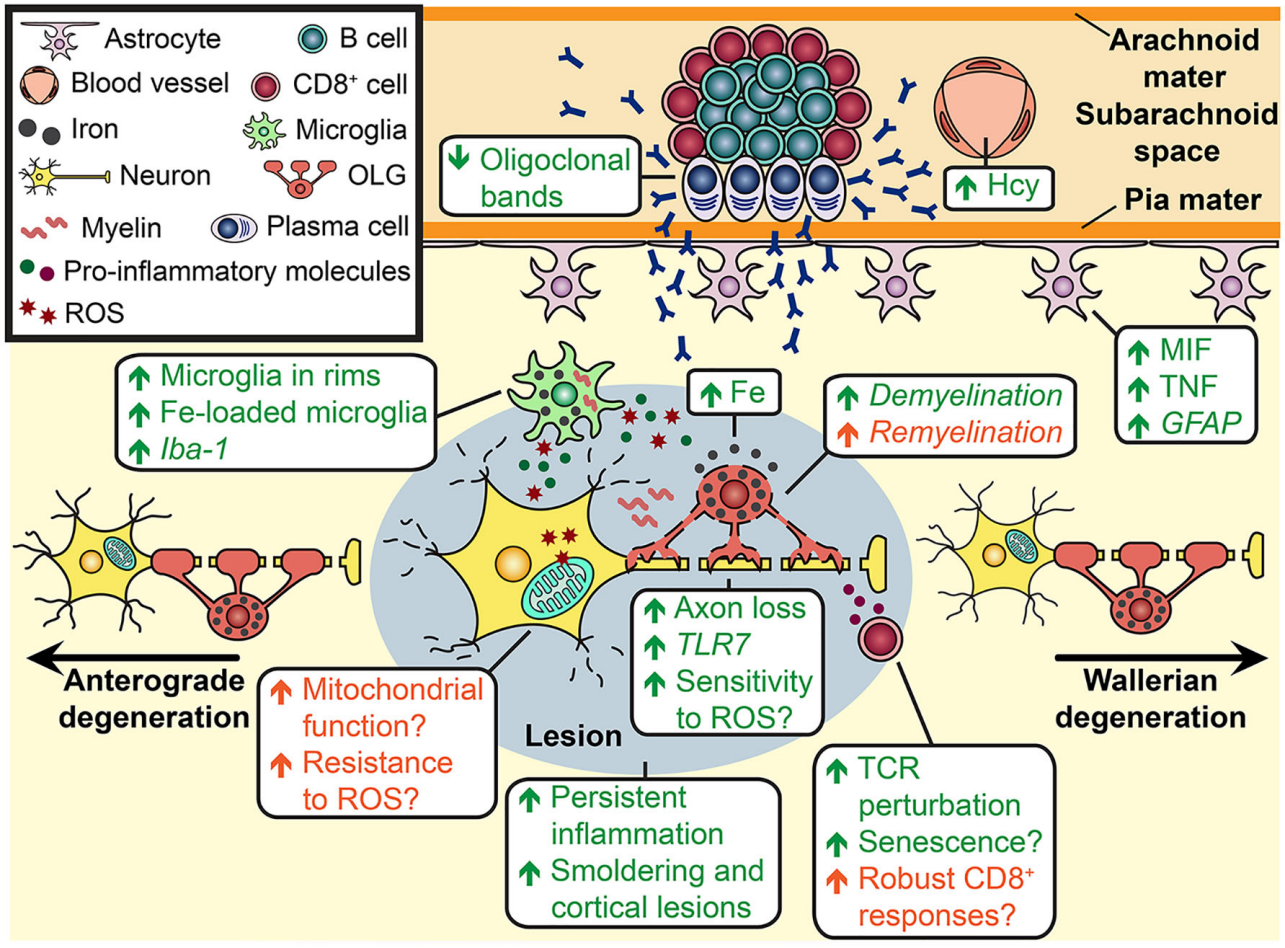
Cellular mechanisms that may be involved in sex differences in MS progression. In progressive MS, peripheral immune cells peripharl immue cells reside in the perivascular spaces at the edge of WM lesions (indicated by a *grey oval*) and in ectopic follicle-like structures in the subarachnoid space. T cells secrete pro-inflammatory cytokines, which can activate neighbouring microglia to produce other pro-inflammatory cytokines such as TNF and reactive oxygen species (ROS). ROS production leads to mitochondrial damage in neurons, which can trigger calcium-dependent proteases and damage enzymes in the mitochondrial respiratory chain. The latter increases mitochondrial ROS, which elicits even more oxidative damage. Also, the immune attack mediated by pro-inflammatory cytokines, antibody/complement and cytotoxic T cells causes the loss of oligodendrocytes (OL), leaving the axons even more vulnerable to damage. The death of OLs leads to the release of iron, which can convert to the ferrous form that reacts with ROS to produce the hydroxyl molecule. Iron is detected as deposits in the extracellular space and is taken up by microglia. Pro-inflammatory cytokines produced by microglia also activate astrocytes to adopt a neurotoxic phenotype. Furthermore, axon damage results in anterograde and Wallerian degeneration in the neuron. Sex differences have been detected in a number of these immune cells and processes. The *green text* indicates cellular mechanisms that are more prominent in males and the *orange text* indicates cellular mechanisms that are more prominent in females in MS (*regular font*) or in EAE (*italic font*). Sex differences that have been described in other disease models (not in MS and EAE) are displayed with a question mark. For example, males T cells have a higher propensity to be pro-inflammatory and reside near activated microglia at the rims of aged WM lesions. Male OLs also carry a higher iron load, which is released upon immune-mediated damage. Microglia loaded with iron are detected at an increased frequency at the rims of WM lesions of male MS patients. There is evidence of greater perturbation in the male CD8^+^ T cell repertoire suggesting greater clonal expansion of these cells with MS; this can lead to the T cells becoming pro-inflammatory. In addition, it has been consistently reported across a number of disease models that astrogliosis is higher in males and the production of the astrocyte factor MIF is increased uniquely in males with MS. Homocysteine, which is a neurotoxic factor, is present at higher levels in the blood of male MS patients. In contrast, females are more likely to exhibit overall greater CD8^+^ T cell responses which may translate into greater anti-viral immunity. This could counter MS progression if EBV is re-activated and is driving this process.

There is more neurodegeneration in males than in females with MS that explains the more rapid physical and cognitive decline in this sex. There are a number of biological mechanisms that may contribute to this more severe neurodegeneration in men (**Figure 1**). First, inflammation in the WM plaque persists longer in males. Males also develop a higher number of cortical demyelinating lesions. This may be due to individual T cells being more pro-inflammatory or microglia and astrocytes becoming more reactive in the males, as evidenced by greater TNF and MIF expression at WM lesion sites in males. Iron load is higher in men in the deep grey matter regions and also accumulates more substantially at the rim of WM lesions in males, which may contribute to greater oxidative damage and inflammation. Furthermore, myelin repair mechanisms are more efficient in female rodents. Finally, there is strong evidence that neurons of males have a greater vulnerability to oxidative damage that may relate to sex differences in mitochondria respiration and antioxidant defense mechanisms. Future studies should focus on defining the basis for the increased smoldering inflammation seen in male WM lesions as well as better explore the impact of environmental influences such as EBV, diet, the gut microbiota, obesity, and cigarette smoking in amplifying these sex differences in neurological progression.

## Data availability statement

The original contributions presented in the study are included in the article/supplementary material. Further inquiries can be directed to the corresponding author.

## Author contributions

NA-S and SD both contributed to the research and writing of this manuscript. All authors contributed to the article and approved the submitted version.
